# Integrated genome-based probiotic relevance and safety evaluation of *Lactobacillus reuteri* PNW1

**DOI:** 10.1371/journal.pone.0235873

**Published:** 2020-07-20

**Authors:** Kazeem Adekunle Alayande, Olayinka Ayobami Aiyegoro, Thizwilondi Michael Nengwekhulu, Lebogang Katata-Seru, Collins Njie Ateba

**Affiliations:** 1 Antibiotic Resistance and Phage Biocontrol Research Group, Department of Microbiology, Faculty of Natural and Agricultural Sciences, North-West University, Mmabatho, South Africa; 2 Food Security and Safety Niche Area, Faculty of Natural and Agricultural Sciences, North-West University, Mmabatho, South Africa; 3 Gastrointestinal Microbiology and Biotechnology Division, Agricultural Research Council, Animal Production Institute, Irene, South Africa; 4 Department of Chemistry, Faculty of Natural and Agricultural Sciences, North West University, Mmabatho, South Africa; Cornell University, UNITED STATES

## Abstract

This study evaluates whole-genome sequence of *Lactobacillus reuteri* PNW1 and identifies its safety genes that may qualify it as a putative probiotic. It further extracted the bacteriocin produced by the strain and tested its effectiveness against pathogenic STEC *E*. *coli* O177. The genomic DNA was sequenced on illuminal Miseq instrument and the sequenced data was assessed for quality reads before assembled with SPAdes. The draft assembly was annotated with Prokaryotic Genome Annotation Pipeline (PGAP) and Rapid Annotations using Subsystems Technology (RAST). Further downstream analyses were carried out using appropriate bioinformatic tools. Production of biogenic amines was biochemically confirmed through HPLC analysis. The assembled genome was 2,430,215 bp long in 420 contigs with 39% G+C content. Among all known genes, putatively responsible for the production of toxic biochemicals, only arginine deiminase (EC3.5.3.6) was spotted. Coding sequences (CDS) putative for D-lactate dehydrogenase (EC1.1.1.28), L-lactate dehydrogenase (EC1.1.1.27) and bacteriocin helveticin J were found within the genome together with plethora of other probiotic important genes. The strain harbours only resistant genes putative for Lincosamide (*lnuC*) and Tetracycline resistant genes (*tetW*). There was no hit found for virulence factors and probability of the strain being a human pathogen was zero. Two intact prophage regions were detected within the genome of *L*. *reuteri* PNW1 and nine CDS were identified for insertion sequence by OASIS which are belong to seven different families. Five putative CDS were identified for the CRISPR, each associated with Cas genes. Maximum zone of inhibition exhibited by the bacteriocin produced *L*. *reuteri* PNW1 is 20.0±1.00 mm (crude) and 23.3±1.15 mm (at 0.25 mg/ml) after being partially purified. With the strain predicted as non-human pathogen, coupled with many other identified desired features, *L*. *reuteri* PNW1 stands a chance of making good and safe candidates for probiotic, though further *in-vivo* investigations are still necessary.

## Introduction

Awareness on the role of health functional food in the gut microbial ecosystem [1]; and its impact on the well-being of humans and animals is fast spreading among consumers. Probiotics are among the fastest-growing health functional foods [2] and the increasing interest in them is responsible for the concerted efforts towards the development of probiotic-based products [3, 4]. Probiotics are live organisms, often bacteria, with beneficial effect on health besides the usual nutritional advantage when consumed in sufficient amounts [5]. They are widely use in the prevention and treatment of several kinds of infectious diseases, with substantial scientific evidence supporting their potency in clinical applications [6]. Evidence from scientific reports have linked their beneficial effect in the gastrointestinal environment to modulation of immune and certain physiological systems, hence, reducing the incidence of diseases [7] and thereby classified among popular bioactive agents in formulating health functional foods [8].

Effectiveness of probiotics is strain specific and cannot be generalised [9]; the same applies to their safety characteristics. Notwithstanding the clinical efficacy of a probiotic agent, safety of the strain involved must be assured; therefore, evaluation of the risk factors of a specific strain must be adequately scrutinized [10]. Hence, a number of efficacy and safety assessment protocols have been recommended, by the experts, for a putative probiotic candidate before confirmation and acceptance for public consumption. Although majority of commonly used organisms as probiotics are regarded as safe, however, the continuous emergence of new strains, partly due to environmental pressure, makes proper and thorough safety screening for every novel strain inevitable. No new strain should be assumed of sharing the same documented safety history with preexisting ones [11].

Moreover, studies have revealed that bioactive secondary metabolites produced by many probiotic agents have implications on bacterial community interaction and, consequently, attenuate the virulent markers on several pathogens [12]. Bacteriocins; the small heterogeneous bioactive peptides synthesized by probiotic bacteria against neighbouring competitive pathogens [13]; have been widely investigated in relation to their desirable impact on the health of human beings and animals [14]. Most bacteriocins are highly specific in their clinical responsibilities, commonly operate distinct mechanisms of action away from the conventional synthetic antimicrobials and are susceptible to genetic manipulation aimed at desired traits due to their peptide nature [15]. In addition, the specificity of bacteriocins allows room for controlled target against pathogens with little or no undesirable effects on the gut microbial ecosystem, though there are other strains that secrete broad spectrum bacteriocins, which can be applied in the situation of infections with difficult-to-identify causative agents [16, 17]. This study is thus, designed to assess the complete genome of *Lactobacillus reuteri* PNW1 for its probiotic potential; possible associated risk factors; and *in vitro* effectiveness of the bacteriocin produced by the test strain.

## Materials and methods

### Ethics statement

All procedures involved in this study complied with relevant legislation regarding protection of animal welfare and were approved by the Agricultural Research Council, Animal Production Institute Ethics Committee.

Field permit number: APIEC13/008

### Extraction of genomic DNA

The candidate probiotic bacterial strain was isolated from the gastrointestinal tracts of compassionately sacrificed weaned piglets of the indigenous South African Windsnyer pig breed (APIEC13/008). The organism was cultured in de Man-Rogosa-Sharpe broth (Oxoid, UK), under strict anaerobic conditions and incubated at 37°C for 24 hours in an anaerobic jar provided with an AnaeroGen system (Thermofisher, UK). The culture was later washed twice in phosphate buffer saline (PBS) and centrifuge at 6000 rpm for 5 minutes each time. Bacterial genomic DNA was extracted with a DNA extraction kit (Zymo Research, USA), in accordance with the manufacturer’s instructions. The degree of purity and concentration of the extracted DNA were determined using a nanodrop spectrophotometer (NanoDrop 2000, ThermoFisher). The genomic DNA material was kept at −20°C for further use.

### 16S rRNA identification of the isolates

Identification of isolates was carried out through PCR amplification of the 16S rRNA region. Genomic DNA was used as the template (16S rDNA) with universal primers fD1 (5’-AGAGTTTGATCCTGGCTCAG-3’) and rD1 (5’-AAGGAGGTGATCCAGCC-3’). The PCR conditions included a first step of 95°C for 2 minutes and 40 cycles of 95°C for 30 seconds (denaturation), followed by 45°C for 30 seconds (annealing) and 72°C for 30 seconds (extension) with a final extension step of 72°C for 7 minutes [18]. Amplicon was confirmed by gel-electrophoresis for 45 minutes at 60 V in 1% (w/v) agarose gel with ethidium bromide (0.5 mg/ml) in 0.5TAE (Tris-Acetate-EDTA) buffer (pH 8.0) and then observed under UV light.

The PCR amplicon was sequenced and the nucleotides were aligned with the National Centre of Biotechnology Information (NCBI) database, using BLAST algorithm. The partial sequenced data (1247 bp) was submitted to the GenBank data base and accession number (MK123483) received for the isolates (https://www.ncbi.nlm.nih.gov/nuccore/MK123483.1/).

### Whole Genome Sequence (WGS) of the isolates

The genome was prepared using an Illumina Nextera DNA Flex library prep kit, and the run performed on an Illumina MiSeq Platform1 system at the Agricultural Research Council, Pretoria, South Africa. Condition of the run and details of statistical analysis summarizing basic characteristics of the read contigs and draft assembly have been mentioned in our previous study [19].

### Identification of important probiotic genes in the draft genome assembly

Coding sequences putative for important probiotic genes were determined through functional annotation which was generated using the NCBI Prokaryotic Genome Annotation Pipeline (PGAP) v. 4.6 [20, 21] and Rapid Annotations using Subsystems Technology (RAST) [22].

### Identification of antimicrobial resistance genes

A quick identification of protein-encoding sequences putative for antimicrobial resistance genes acquired within the draft genome was performed using ResFinder v. 3.1 [23] and Comprehensive Antibiotic Resistance Database (CARD) v. RGI 5.1.0, CARD 3.0.7 [24]. Data from the functional annotation through RAST were also manually searched for resistance against a number of clinically important antimicrobials.

### Identification of virulent determinant genes

Putative virulence determinant and pathogenicity of strain were determined using VirulenceFinder v. 2.0 [55] and PathogenFinder v. 1.1 [25] respectively. Manual searches for protein-encoding genes related to virulence in bacteria were also employed based on the functional annotation from the RAST platform. Factors considered included the following: sex pheromones; gelatinase; cytolysin; hyaluronidase; aggregation substance; enterococcal surface protein; endocarditis antigen; adhesine of collagen; and integration factors.

### Identification of prophage, transposase and other Insertion Sequences (IS) within the genome

Rapid identification and annotation of prophage sequences was determined using the Phage Search Tool Enhanced Release (PHASTER) [26]. The protein-encoding genes for transposase were manually searched within the functional annotation data generated from the NCBI-PGAP and RAST platform. The Genome was searched for insertion sequences (IS) using ISfinder search tool, Insertion Sequence Semi-Automatic Genome Annotation (ISsaga V. 2.0) [27] and Optimised Annotation System for Insertion Sequences (OASIS) [28].

### Identification of CRISPR−Cas sequences within the genome

Coding sequences for Clustered Regularly Interspaced Short Palindromic Repeats (CRISPR) and CRISPR-associated genes (Cas) were indentified using CRISPRCasFinder v. 1.1.2—I2BC [51] and manual searches through the functional annotation generated from RAST database.

### Determination of toxic biochemical and associated genes

#### Genome-based

Protein-encoding sequences putative for enzymes directly related to the production of biogenic amines, such as histidine decarboxylase, tyrosine decarboxylase, ornithine decarboxylase, agmatine dehydrolase, L-lysine decarboxylase and agmatine deiminase pathway, were manually investigated through functional annotation from NCBI-PGAP and RAST platform. Further genes involved in the production of toxins, such as haemolysin and cytotoxin K; and those involved in the production of lipopeptides, such as fengygin, surfactins and lychenisin, were also investigated from the same database [29].

#### Biochemical extraction and determination of biogenic amines

Bacterial cells were sub-cultured in de Man-Rogosa-Sharpe broth supplemented with amino acid supplements, which included L-histidine mono-hydrochloride (2.5 g/L), L-tyrosine disodium salt (2.5 g/L), L-ornithine mono-hydrochloride (2.5 g/L), L-lysine mono-hydrochloride (2.5 g/L) and agmatine sulfate salt (1g/L) (Sigma-Aldrich, Germany). The cultures were incubated, under strict anaerobic conditions, in an anaerobic jar provided with anaerocult A (Merk, Germany), at 37°C for 3 days without agitation [29, 30].

Extraction and determination of histamine, tyramine, putrescine, cadaverin and agmatine were performed as previously described by Singracha *et al*. [31]. Bacterial culture (50 ml) was centrifuged at 10,000 rpm for 10 minutes at 10°C and the supernatant (5 ml) extracted in 25 ml of 0.4M perchloric acid and then transferred into a screw-capped bottle. Then, 1 ml of crude extract was added with 10 μl of the internal standard (1, 7- diaminoheptane), 200 μl of 2 M sodium hydroxide (NaOH), 300 μl of saturated sodium bicarbonate (NaHCO_3_) and 1000 μl of dansyl chloride (10 mg/ml in acetone) and then vortexed. The mixture was incubated at 70°C for 30 minutes and excess dansyl chloride was precipitated with 100 μl of ammonium hydroxide (NH_4_OH, 30%). The supernatant was adjusted to 50 ml with acetonitrile and filtered through 0.45 μm Polytetrafluoroethylene membrane filter and the filtrate was kept at -28°C prior to HPLC analysis. Determination of biogenic amines from the extract was carried out using High Performance Liquid Chromatography (PerkinElmer Altus, A-10 PDA Detector) with a C18 RP-HPLC column (Analytical C18 Column 100 x 4.6 mm, 5 μm particle size). Deionized water and acetonitrile were used as the gradient elution system at a flow rate of 0.5 ml/min. Elution gradient of 65% acetonitrile was used at 0 minute, increased to 70% at 5 minutes and 100% at 20 minutes and then 65% at 25 minutes. Histamine, tyramine, putrescine, L-agmatine and cadaverine (Sigma-Aldrich, Germany) were used as standards.

### Cultivation of *L*. *reuteri* PNW1 for bacteriocins production

The probiotic bacterial isolate was sub-cultured into De Man Rogosa Sharpe (MRS) broth under complete anaerobic condition and incubated at 37°C for 24 hours in an anaerobic jar supplemented with AnaeroGen system (Thermofisher, UK). The culture was then centrifuged at 10,000 rpm for 10 minutes and the pH of the supernatant adjusted to 6.5. Thereafter, the supernatant was filtered through 0.22 μm syringe filter (Millex syringe filters, Sigma Aldrich) and the filtrate (crude bacteriocin) was tested for antimicrobial potential and thereafter kept in the refrigerator for further analysis [32].

### Partial purification of crude bacteriocins produced by the *L*. *reuteri* PNW1

Purification of crude bacteriocins was done as previously described [33, 34]. One liter of crude bacteriocins was partially purified by precipitation with 80% saturated ammonium sulphate and left overnight under a temperature condition of 4°C with regular stirring. The precipitates were collected and then re-suspended in 0.1 M phosphate buffer solution (pH 7). This was, thereafter, extracted in a mixture of chloroform and methanol (2:1, v/v) and then lyophilized.

### Determination of the antimicrobial potential of the bacteriocin produced by *L*. *reuteri* PNW1

Agar well diffusion method was used to determine the antimicrobial potential of bacteriocin against pathogenic STEC *Escherichia coli* O177 [33]. Two different strains of pathogenic *E*. *coli* O177 were collected from culture collection of the Molecular Microbiology Research Laboratory, North-West University Mahikeng campus, South Africa. A 24 h old nutrient broth culture of each of the test strain was standardised (0.5 McFarland standard). Then 100 μl of the standard inoculums was inoculated into molten (50^**°**^C) sterile freshly prepared Muller Hinton agar, gently mixed and then poured into a sterile Petri dish. The mixture was allowed to set before boring wells into the agar, using 6 mm cork borer. The wells were carefully filled with the crude extracted bacteriocin and not allowed to spill. The plates were incubated at 37^**°**^C for 24 hours. After this process, the clear zone of inhibition was measured. The experiment was carried out in three replicates.

## Results

**Fig 1:**
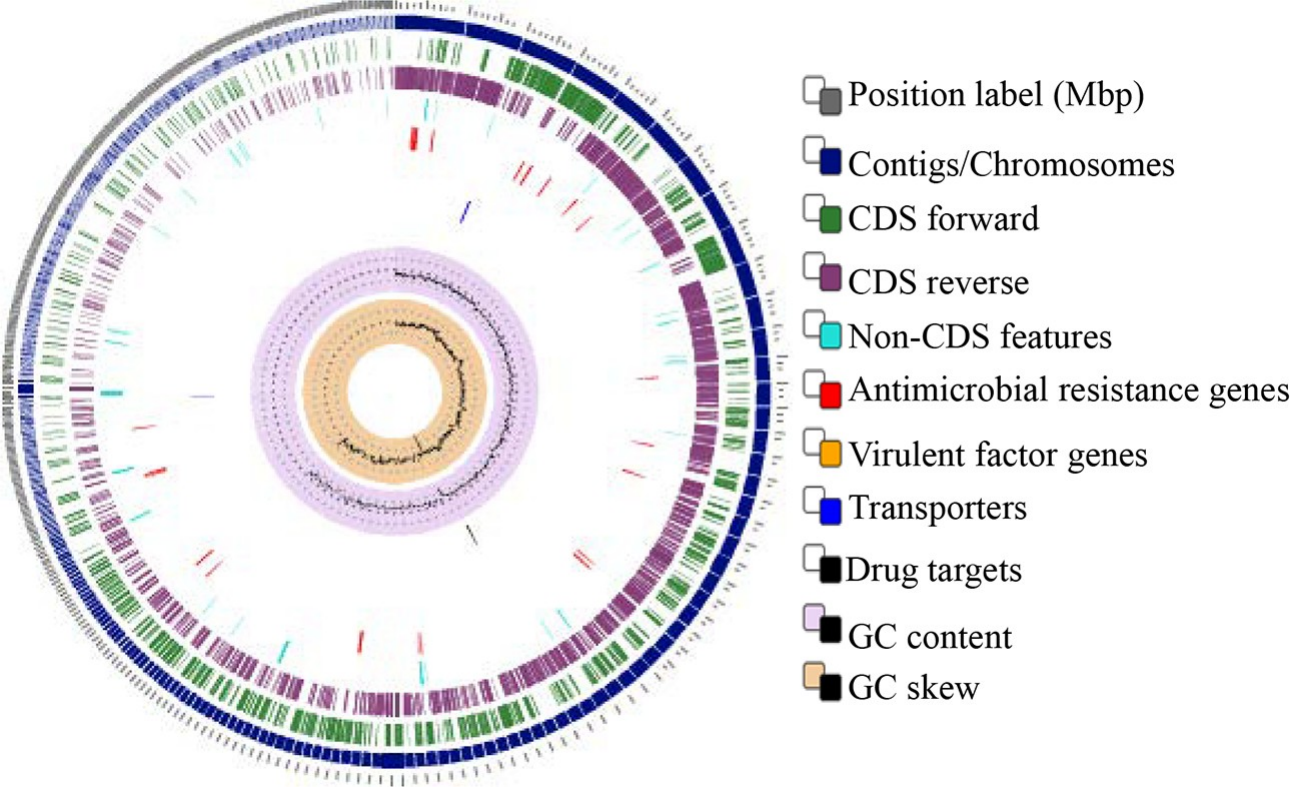
Circular genome mapping of the *L*. *reuteri* PNW1. The circular genome was generated with PATRIC sever 3.5.43.

The entire genome of *L*. *reuteri* PNW1 was sequenced to assess the probiotic relevance and safety features of the strain. The circular representation of the complete genome is shown in Fig 1. The whole-genome shotgun project was deposited at DDBJ/ENA/GenBank under accession number RJWE00000000. The version described in this study is RJWE01000000 with SRA accession number PRJNA504734.

### Probiotic potential of the strain

A total number of four (4) coding sequences (CDS) predictably encoding for D-lactate dehydrogenase (EC 1.1.1.28) and eight (8) for L-lactate dehydrogenase (EC 1.1.1.27); both of which are responsible for production of lactic acids; were found on different loci within the genome (Table 1). The coding sequence putative for a bioactive peptide predicted to be bacteriocin helveticin J (318 bp, forward strand) and another CDS for a therapeutically useful peptide, S-ribosylhomocysteinelyase (EC 4.4.1.21) @Autoinducer-2 production protein *LuxS* (477 bp, reverse strand) were both confirmed present within the genome.

**Table 1:** Identified protein-encoding genes putative for lactic acids production by *L*. *reuteri* PNW1.

Gene function	Contig no	Fragment position	Sizes (bp)	DNA strand
D-lactate dehydrogenase (EC 1.1.1.28)	9	27872–26877	995	Reverse
	34	19146–20150	1004	Forward
	84	4462–3470	992	Reverse
	121	204–1196	992	Forward
L-lactate dehydrogenase (EC 1.1.1.27)	1	106437–105502	931	Reverse
	3	15382–16356	974	Forward
	5	66551–67255	704	Forward
	5	67218–67490	272	Forward
	7	31496–30564	932	Reverse
	8	28064–29023	959	Forward
	16	29593–28670	923	Reverse
	38	13817–12867	950	Reverse

Key: ID = Identity, EC = Enzyme commission number

Moreover, a number of different coding sequences putatively involved in adhesion of the *L*. *reuteri* PNW1 to the surrounding epithelial tissue were identified within the genome. The identified gene with adhesive functions include, Antiadhesin Pls; Sortase A, LPXTG; exopolysaccharides (EPS) cluster; ATP synthase epsilon chain; and DNA polymerase III, epsilon subunit related 3'-5' exonuclease (Table 2). A quick search for desired stress tolerance feature of *L*. *reuteri* PNW1 reveals the presence of CDS predictably encoding for DNA protection during starvation protein which was found on two different loci within the genome assembly. These were located on contig number 21 and 53 while both fragments measured 468 and 546 bp long respectively on the forward DNA strand. Another stress resistant gene putatively encoding for Phosphate starvation-inducible protein PhoH, predicted ATPase, with CBSS-56780.10.peg.1536; was also detected on the contig number 8 and the fragment is 1008 bp long located on the reverse DNA strand.

**Table 2:** Identified protein-encoding genes putative for adhesion by *L*. *reuteri* PNW1.

Gene function	Contig no	Fragment position	Sizes (bp)	DNA strand
AntiadhesinPls, binding to squamous nasal epithelial cells	14	16625–15555	1070	Reverse
Sortase A, LPXTG	19	21517–20813	705	Reverse
Tyrosine-protein kinase transmembrane modulator EpsC	16	981–352	630	Reverse
Tyrosine-protein kinase EpsD (EC 2.7.10.2)	40	2439–1693	747	Reverse
Tyrosine-protein kinase transmembrane modulator EpsC	40	3327–2452	876	Reverse
ATP synthase epsilon chain (EC 3.6.3.14)	27	5543–5112	432	Reserve
DNA polymerase III, epsilon subunit related 3'-5' exonuclease	49	12311–11772	540	Reverse

Among the important probiotic features identified in the *L*. *reuteri* PNW1 are five different CDS in connection with improving metabolism of the host. Three of the CDS were predicted for the same enzyme, Poly (glycerol-phosphate) alpha-glucosyltransferase (EC 2.4.1.52); and two of which occurred on the same loci with contig identity NODE_72_length_9092_cov_690.969994 (Table 3). Additionally, a plethora of protein-encoding sequences putative for extracellular hydrolytic enzymes lipase and protease were found within the genome of the study strain while none was identified for amylase and cellulase.

**Table 3:** Identified protein-encoding genes putatively involved in active metabolism in the host.

Gene function	Contig no	Fragment position	Sizes (bp)	DNA strand
Poly (glycerol-phosphate) alpha-glucosyltransferase (EC 2.4.1.52)	5	47264–48787	1524	Forward
	72	8405–6864	1542	Reverse
	72	6862–5360	1503	Reverse
Beta-1, 3-glucosyltransferase	95	3365–4378	1014	Forward
Xylose isomerase domain protein TIM barrel	103	1669–2508	840	Forward

### Antimicrobial resistance and pathogenicity of the strain

Separate searches through the Centre for Genomic Epidemiology database ResFinder and Comprehensive Antibiotic Resistance Database (CARD) for acquired antimicrobial resistance encoding genes revealed that *Lactocbacillus reuteri* PNW1 only harbours resistant genes putative for lincosamide nucleotidyltransferase (*lnuC*) and Tetracycline-resistant ribosomal protection protein (*tetW*) that confer resistance to lincosamide and tetracycline respectively. Manual searches through the functional annotation data also revealed the same results (Table 4).

**Table 4:** Identified antimicrobial resistance genes harbours by *L*. *reuteri* PNW1.

Gene function	Contig no	Fragment position	Sizes (bp)	DNA strand
Lincosamide nucleotidyltransferase (*lnuC*)	126	1150–1644	495	Forward
Tetracycline resistance and Ribosomal protection type (*TetW*)	89	1027–368	660	Reverse
	89	2289–1048	1242	Reverse
Ribosome protection-type Tetracycline resistance-related proteins, group 2	34	1887–3815	1929	Forward

Inquiry on pathogenicity of the strain using PathogenFinder tool host by the Centre for Genomic Epidemiology revealed that, calculated Matched Pathogenic Families for *L*. *reuteri* PNW1 was 0; the Matched Not Pathogenic Families was 343; and probability of the strain being a human pathogen was 0.217. Thus, the strain was predicted as a non-human pathogen. Moreover, VirulenceFinder used to determine possibility of virulence factors within the genome of the strain returned no hit for any virulence determinant. The entire genome assembly was further searched manually through the functional annotation. Nine notable virulence determinants were manually searched and none of them was found within the genome.

### Stability of the genome of *L*. *reuteri* PNW1

**Fig 2:**
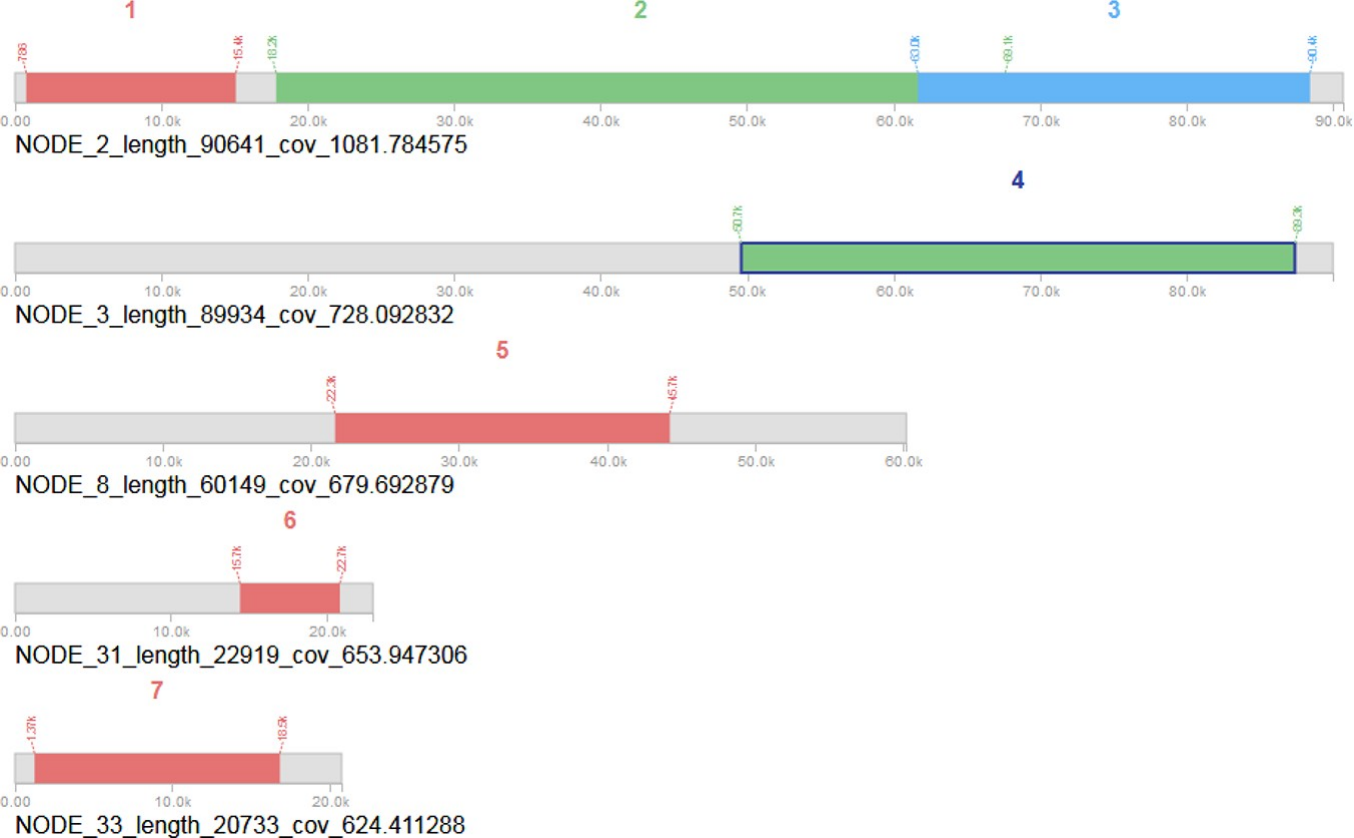
Genome mapping of the *L*. *reuteri* PNW1 showing regions and positions occupied by different types of prophage. Prophage identified as intact (⁑), incomplete (⁑) and questionable (⁑).

A number of Mobile Genetic Elements (MGE), such as prophage, tranposease and other insertion sequences was identified within the genomes of the *Lactobacillus reuteri* PNW1. Seven predicted prophage regions were identified within the genome of the strain. Two among the regions were identified as intact; four designated as incomplete; and the remaining one region was identified as questionable. The two intact prophages are found on contigs number 2 and 3 (Fig 2).

**Fig 3:**
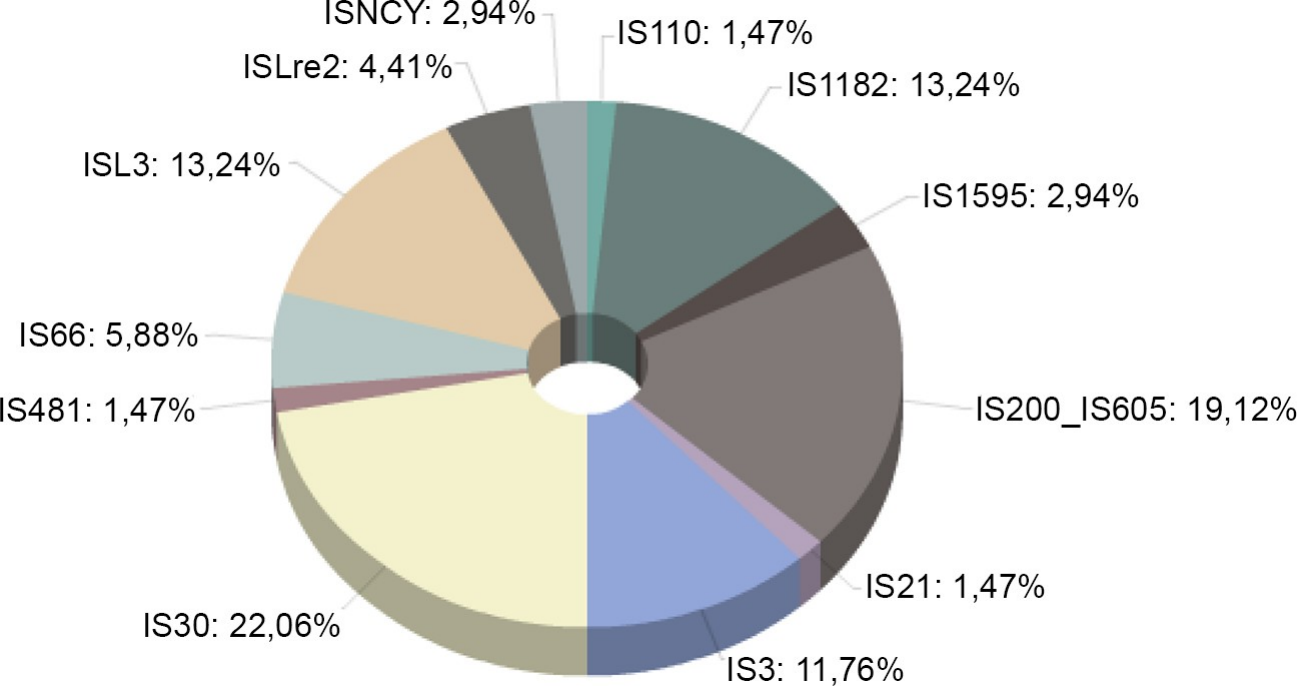
Genome mapping of the *L*. *reuteri* PNW1 showing distribution of roughly predicted IS family within the genome using ISsaga v 2.0.

Determination and classification of transposase-encoding genes and other putative insertion sequences (IS) and associated proteins within the genome of *L*. *reuteri* PNW1 by OASIS reveals a total number of fourteen CDS predicted for IS. Out of which nine different IS were confirmed and distributed into seven different families. The IS families identified by OASIS are, IS1182; IS1595; IS200—IS605; IS21; IS30; IS3; and ISLre2. On the other hand, when ISsaga search tools was employed, 21 putative complete ORF were roughly identified as IS and distributed into 12 different IS families (Fig 3). Only five of these were completely annotated and provided with details of the sources, functions and insertion sites in accordance with the ISfinder (Table 5). The remaining ORF was confirmed through blastx on NCBI data base. When the *L*. *reuteri* PNW1 genome was searched for putative coding sequences for CRISPR−Cas sequences, five CDS for the Clustered Regularly Interspaced Short Palindromic Repeats (CRISPR) were detected by the CRISPRCasFinder. Each of the CDS contains single associated cas-gene or repeat consensus and single spacer gene (Table 6).

**Table 5:** Semi-automatic complete annotation of the IS found within *L*. *reuteri* PNW1 genome using the ISfinder search tool.

IS Name	IS Family	IS Origin	Length (bp)	ORF Function
ISLre2	ISLre2	*Lactobacillus reuteri*	1570 bp	Transposase
ISLac1	IS1182	*Lactobacillus acidophilus*	1833 bp	Transposase
ISCco2	IS1595	*Campylobacter coli*	7852 bp	1. Transposase
				2. Passenger Gene (Streptothricin Adenyltransferase, Streptothricin acetyltransferase)
ISSag10	IS1595	*Streptococcus agalactiae*	1724 bp	1. Transposase
				2. Passenger Gene (O-lincosamide nucleotidyltransferase)
IS2001	ISL3	*Bifidobacterium lactis*	1406 bp	Transposase

Key: ORF = open reading frame

**Table 6:** Putative CRISPR-Cas sequences found within *L*. *reuteri* PNW1 genome.

Contigs no	Fragment position	Spacers count	Repeat consensus/cas-genes
56	1–133	1	ACTGCAGATAGTGGTCAGCCCAACAACGCTCAAACCAAAC
56	12460–12586	1	GGTCAGCCCAACAACGCTCAAACCAAACCTGGTA
136	1173–1253	1	GCCGAAGACATGAGACAACTTATTT
169	134–224	1	ATCGATTCCGCGCGTGACATGGGCGCCAA
410	31–114	1	GCCCCTTCTGCGTAAAAAGAGAC

### Toxic biochemicals

Functional annotation of the entire genome of *L*. *reuteri* PNW1 revealed that the *L*. *reuteri* PNW1 does not possess any protein-encoding gene involved in the production of biogenic amines; which are products of decarboxylation of specific free amino acids; with the exception of CDS putative for arginine deiminase and no other toxin was identified. Seven different coding sequences related to arginine deiminase pathway with functional ornithine degradation were found in the genome of *L*. *reuteri* PNWI (Table 7).

**Table 7:** Identified protein-coding genes putative for arginine deiminase pathway.

Gene function	Contig no	Fragment position	Size (pb)	DNA strand
Arginine deiminase (EC 3.5.3.6)	63	4969–3737	1233	Reverse
Ornithine carbamoyltransferase (EC 2.1.3.3), invloved in arginine deiminase pathway	31	1553–2560	1008	Forward
Arginine/Ornithine antiporterArcD	63	3144–1723	1422	Reverse
	63	1665–268	1398	Reverse
	25	9043–7526	1518	Reverse
	57	9013–10566	1554	Forward
	16	18621–20042	1422	Forward

Analysis of the HPLC carried out to determine possible production of biogenic amines by the isolates revealed the presence of putrescine as the only biogenic amine produced. The putrescine produced by *L*. *reuteri* PNW1 was eluted within a retention time of 2. 334 min at a flow rate of 0.5 ml/min. When compared, the putrescine used as standard reference eluted at a retention time of 2.436 min under the same flow rate of 0.5 ml/min.

### Susceptibility test

**Fig 4:**
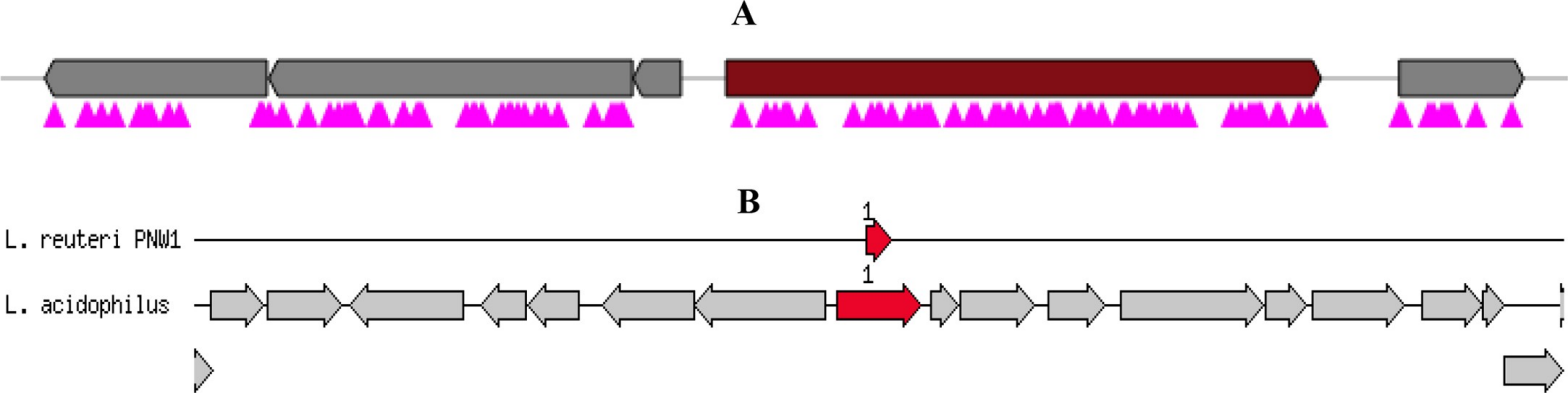
A. Gene cluster showing the position of biosynthetic bacteriocin. Core biosynthetic (bacteriocin) genes (■), other genes (■), TTA codon (■)—antiSMASH v. 4.2.0. B. Annotation diagram showing the location of bacteriocin helveticin J. Bacteriocin helveticin J ($\overset{1}{➡}$) found at node 430 and 318 bp long on the +ve strand—RAST sever with SEED viewer v.2.0.

After the genomic confirmation of the bacteriocin through functional annotation and *in silico* analysis of the secondary metabolites (Fig 4A and 4B), the bioactive peptide was extracted from the isolate. The crude and partially purified bioactive peptide (bacteriocin) produced by the *L*. *reuteri* PNW1 was tested against 2 strains of pathogenic *E*. *coli* O177. Both test strains were susceptible to the bacteriocins. The zone of inhibition exhibited by the crude bacteriocin ranged between 18.7±0.58 and 20.0±1.00 mm while that of the partially purified bacteriocin ranged between 22.0±0.00 and 23.3±1.15 mm (Table 8).

**Table 8:** Susceptibility test of crude and partially purified bacteriocins produced *L*. *reuteri* PNW1.

	Zones of inhibition (mm)*	
Organism	BP1	BP1_PR (0.25 mg/ml)
*E*. *coli* C1	18.7±0.58	23.3±1.15
*E*. *coli* C2	20.0±1.00	22.0±0.00

Key: BP1 –Crude bioactive peptide; BP1_PR–Partially purified bioactive peptide;

* - Mean values of three replicates

## Discussion

Complete genome analysis and functional annotation of the *Lactobacillus reuteri* PNW1 revealed the presence of several genes within the genome assembly, which are important for probiotic efficacy. Several scientific reports have indicated that bioactive secondary metabolites produced by many probiotic agents, have implications on bacterial community interaction and, consequently, attenuate virulent markers on a number of pathogens [35, 36, 37, 38]. For instance, lactic acids produced by Lactic acid bacteria hinder the survival of neighbouring pathogens and also inactivates human immunodeficiency virus by increasing acidity of the surrounding environment [12]. A total of 12 coding sequences were found in the genome of *L*. *reuteri* PNW1, predictably encoding for both D-lactate dehydrogenase (EC 1.1.1.28) and L-lactate dehydrogenase (EC 1.1.1.27) which are responsible for lactic acids production. In addition, a promising probiotic candidate with inherent ability for producing bioactive peptides and hydrogen peroxide would be an added advantage in antagonising neighbouring pathogens within the gut ecosystem [39]. The *in silico* analysis of the entire genome for putative secondary metabolites, coupled with the functional annotation of the coding sequences within the *L*. *reuteri* PNW1 genome assembly, revealed the occurrence of CDS for bacteriocin helveticin J and S-ribosylhomocysteinelyase (EC 4.4.1.21) @Autoinducer-2 production protein *LuxS*, a protein involved in the secretion of bioactive peptides.

Adhesion of a probiotic candidate to the epithelia cells and mucus layer is a desired trait for a probiotic. Adhesive characteristics identified in *L*. *reuteri* PNW1will provide ample opportunity for stability of the strain and effectively prolong the antagonistic effects against unwanted gut residents, thus aiding in effective colonisation of the gut environment and exclusion of pathogens [40, 41]. For example, exopolysaccharides (EPS) identified in the strain are generally regarded as a food-grade and contribute a great deal to the probiotic-host interactions within the intestinal mucosa and epithelial cells, thus impacting on the strain specificity of probiotic candidates [42]. Many of the EPS have also been attributed to certain essential features of a probiotic such as biofilm formation, immunomodulation, aggregation, antioxidant and antimicrobial potentials [43].

Likewise the anti-adhesive molecules which are specifically interact with the adhesin markers on infecting pathogens, thus preventing colonisation [44]. Presence of the encoding gene for the Antiadhesin Plsin the genome of *L*. *reuteri* PNW1 will greatly improves its clinical effectiveness as a prophylactic agent against infectious diseases. Equally, Sortase dependent proteins are an important group of cell surface proteins in *Lactobacillus* spp. and are responsible for sorting the largest number and various kinds of cell surface proteins, thus playing an important role in adhesion [45, 46]. The LPXTG specific Sortase A enzyme located in the *L*. *reuteri* PNW1 genome is predicted to covalently anchor surface protein precursor to the cell wall, after the precursor has been transferred to the cell membrane [42].

Moreover, certain genes required by a microorganism in order to survive harsh environmental conditions are among the pool of beneficial genes identified in the draft assembly of *L*. *reuteri* PNW1. Two different CDS putative for protecting DNA during starvation protein are found on two different loci. These are important for a probiotic organism to survive adverse condition in the gut. The genome also contains Phosphate starvation-inducible protein PhoH, predicted ATPase in its defense against harsh environments. The CDS is predicted to be involved in Glycyl-tRNA synthetase containing cluster and phosphate metabolisms. The Phosphate starvation-inducible protein PhoH induces the PhoH gene belonging to phosphate regulon and sB-dependent gene belonging to general stress regulon. The sB-dependent general stress proteins (Gsps) are predicted to provide cells with several kinds of non-specific stress tolerance. It is also putatively involved in the protection of DNA, membranes and other proteins against oxidative stress and assists cells to survive extreme conditions, such as heat, osmotic pressure and irregular pH in the environment [47].

Improved metabolic activity is one the important features expected of a probiotic organism to confer on a host. The genome of the *L*. *reuteri* PNW1 harbours a number of genes predicted to be directly involved in improving host metabolism. Xylose isomerase domain protein TIM barrel is one of the genes identified in this capacity. The protein xylose isomerase is responsible for the isomerisation of glucose and pentose sugars in microbial cells [48]. Beta-1,3-glucosyltransferase and Poly (glycerol-phosphate) alpha-glucosyltransferase (EC 2.4.1.52) are also among the genes predicted for the same purpose. Four different coding sequences encoding for Poly (glycerol-phosphate) alpha-glucosyltransferase (EC 2.4.1.52) are found within the genome. β 1-3-glucosyl transferase catalyzes glucose that attached to O-linked fucose through β 1–3 glycosidic linkage on thrombospondin type1 repeats [49].

Considering the plethora of probiotic important genes indentified within the genome of *L*. *reuteri* PNW1, this study went further to assess the safety features of the strain. Quick searches through different databases and functional annotation revealed that *L*. *reuteri* PNW1 harbours resistant genes against Lincosamide and Tetracycline. It is important to note that apassenger gene, which was identified among the insertion sequences in the *L*. *reuteri* PNW1 genome, was found in association with O-lincosamide nucleotidyltransferase. This could suggest the possibility of increased chances of dissemination. However, the co-existing Tetracycline-resistant ribosomal protection protein (*tetW*) was not flanked by any of the annotated insertion sequences.

Moreover, the genomes of the isolate harboured Clustered Regularly Interspaced Short Palindromic Repeats (CRISPR), with associated Cas-gene and spacer. The presence of CRISPR region within a genome limits the spread of antimicrobial resistant genes through obstruction of multiple pathways of horizontal gene transfer [50]. The presence of effective CRISPR regions in *L*. *reuteri* PNW1 genome will equip the strain with sequence specific defence-line against plasmids, insertion sequences and phages [51, 52]. The CRISPR locus, with associated Cas-genes, equally provides the host strain with the potential to defend itself against any incoming extra-chromosomal DNA molecules [53]. This is an indication of stability of the genome of *L*. *reuteri* PNW1 and a very low possibility of the strain to acquire antimicrobial resistant genes since resistance genes are mostly introduced by mobile genetic elements.

Furthermore, among all the protein-encoding sequences related to the production of toxic biochemical, only CDS predicted for arginine deiminase with functional ornithine degradation was found within the genome of the *L*. *reuteri* PNW1.The protein arginine deiminase is a post translational modification enzyme that catalyses hydrolysis of peptidyl-arginine to uncharged non-standard peptidyl-citrulline on relevant proteins such as histones and fibrinogen. This is referred to as citrullination or deamination, that is, post-translational modification of histones, which is the essential building block of chromatin [53, 54]. Up-regulation and over-expression of the arginine deiminase activity, most importantly, class IV, has been observed in several disease conditions such as rheumatoid arthritis, Alzheimer’s disease, multiple sclerosis and cancer [55]. It is equally important to note that protein arginine deiminase class IV is the only isozyme that has been confirmed to be involved in the deimination of histone [54]. Protein arginine deiminase are now being identified with potential involvement in tumor progression [53]. Further study is required to characterize with a view to identifying the class of arginine deiminase which maybe produced by the strain.

Production of the biogenic amine, putrescine by *L*. *reuteri* PNW1 was biochemically confirmed through HPLC analysis. This could be traced to the presence of Ornithine carbamoyltransferase (EC 2.1.3.3), involved in arginine deiminase pathway (Table 7). The enzyme is known to catalyse the reactions of agmatine deiminase and, thus, acting as putrescine synthase which may then convert agmatine [(4-aminobutyl) guanidine] and ornithine into putrescine and citrulline, respectively [56]. Lastly, the extracted bacteriocin from the isolate showed commendable efficacy against the two tested shiga toxin producing *E*. *coli* O177. This could be a pointer towards the use of these organisms as probiotic agents with a focus on controlling post-weaning diarrhoea, which is a major challenge in piggery and dairy farming, coupled with several other physiological benefits of a probiotic agent.

## Conclusion

Considering the plethora of probiotic important genes harboured in the genome of *L*. *reuteri* PNW1, the strain will presumably make a promising candidate for a probiotic supplement. In general, with prediction of the strain as non-human pathogens, coupled with the presence of the CRISPR-Cas regions, which serves to protect and ensure stability of the genomes, together with some other identified factors, the *L*. *reuteri* PNW1 stand a chance of making good and safe candidates for future development of probiotic. However, further *in vivo* investigations in the animal model and human efficacy trials are required in order to effectively assess the level of expression and, possibly, suppression mechanism for the identified gene putative for the production of arginine deiminase.
